# Rabies Epidemiology and Control in Ecuador

**DOI:** 10.5539/gjhs.v8n3p113

**Published:** 2015-07-13

**Authors:** Esteban Ortiz-Prado, Jorge Ponce-Zea, Dario Ramirez, Anna M. Stewart-Ibarra, Luciana Armijos, Jaime Yockteng, Washington B. Cárdenas

**Affiliations:** 1 Universidad de las Americas, Quito, Ecuador; 2 Research and Development Department ENFARMA EP, Quito, Ecuador; 3 Department of Medicine and Center for Global Health and Translational Science, State University of New York Upstate Medical University, Syracuse, NY, USA; 4 Prometeo Program, SENESCYT, Quito, Ecuador; 5 Minister of Public Health, Quito, Ecuador; 6 Laboratorio de Biomedicina, Escuela Superior Politécnica del Litoral, ESPOL, Guayaquil, Ecuador; 7 Instituto Nacional de Investigacion en Salud Publica INSPI, Guayaquil, Ecuador

**Keywords:** Rabies, Rabies vaccine, zoonotic disease, Ecuador, epidemiology

## Abstract

**Objective:**

Describe the epidemiology and the control effort for rabies in Ecuador.

**Methods:**

This observational study included data from the Ecuadorian National Institute of Census and Statistics (INEC), and mortality and morbidity data reported by the Ministry of Public Health and the National Institute for Social Security. We conducted a phylogeny analyses to compare the N gene from the Challenge Virus Standard (CVS) vaccine strain used in Ecuador with published Cosmopolitan, Asian and Sylvatic strains. Descriptive and inferential statistics were used to determine the significance of the data.

**Results:**

In 1996 Ecuador suffered the highest rate of rabies per capita in the Americas, with an incidence rate of 0.56 cases per 100 000 people per year. Human and canine rabies showed a sharp decline until 2012. Between 1994 and 2014, we found a correlation of 0.925 (p<0.01) between annual cases of dog and human rabies. In 2011, there was an epidemic of sylvatic rabies transmitted to people by vampire bats (*Desmodus rotundus*) in the Amazon region, specifically in Morona Santiago, leading to 11 fatalities. Phylogenetic analyses of the CVS vaccine N gene showed an association with urban canine rabies strains (the Cosmopolitan lineage and Asian strains), whereas sylvatic rabies, like those reported in the Amazon region, were found to be grouped in a different clade represented mainly by bat-derived strains.

**Conclusions:**

This study presents the first compilation of epidemiological data on rabies in Ecuador. The incidence of human and canine rabies, also known as urban rabies, has clearly decreased due to massive canine vaccination campaigns. Phylogenetic analysis of the prevailing vaccine used in the country showed a clear separation from bat-derived rabies, the source of recent rabies outbreaks. Efforts are ongoing to develop rabies vaccines that are highly specific to the rabies virus genotype circulating in the region, including sylvatic rabies. These efforts include the implementation of reverse genetics to generate recombinant virus coding for the prevailing glycoprotein gene.

## 1. Introduction

Rabies is a preventable viral disease, typically transmitted through the bite of a rabid animal. The first detailed description of the rabies syndrome, including the long incubation period, is found in Fracastoro's writings during the Dark Ages. Louis Pasteur and Émile Roux, developed the first rabies vaccine in 1885 (Baer, 1991). The causative agent is a ~12 Kb, single-stranded, non-segmented negative sense RNA genome virus that belongs to the Mononegavirales order, Rhabdoviridae family and Lyssavirus genus. The rabies genome encodes five proteins: nucleoprotein (N), phosphoprotein (P), matrix protein (M), glycoprotein (G) and polymerase (L). *Lyssavirus* show a broad antigenic cross reactivity at the nucleocapsid level due to a high sequence conservation of the N gene.

Virus entries are through wounds or by direct contact with mucosal surfaces. The virus replicates on nervous tissues or directly enters into peripheral nerves. Then the rabies virus travels by retrograde axoplasmic flow to the central nervous system, causing ultimately brain disease and death (Archer & Houldcroft, 2014). Rabies is almost inevitably fatal, and death occurs within a few days after symptoms onset. Early signs and symptoms of rabies include fever, headache, weakness anxiety, confusion, paralysis, excitation, hallucinations, agitation, hypersalivation, odynophagia and hydrophobia (Brass, 1994; Susilawathi et al., 2012; Udow, Marrie, & Jackson, 2013). Rabies has the highest case-fatality rate of any currently recognized infectious disease.

Diagnosis of rabies by clinical symptoms is difficult, except when specific disease signs appear as hydrophobia and aerophobia. Laboratory diagnosis can be done *post* *mortem* and *in vitam*; the main techniques are based on antibodies antigen detection, virus isolation and nucleic acid sequences amplification (PCR). Rapid fluorescence focus inhibition test (RFFIT) is the current gold standard serological assay, recommended by the World Health Organization (WHO). Nowadays, rabies affects more than 150 countries worldwide. More than 3.3 billion people live in endemic or enzoonotic regions where approximately 60 000 people die from rabies each year, especially in Asia and Africa (DeMaria, 2014).

The situation in regions like the United States differs considerably, where 1-2 cases per year are reported in the whole population, mainly transmitted by infected bats (Blanton, Palmer, Dyer, & Rupprecht, 2011). Nevertheless, more than 39 000 cases per year of suspicious animal bites that require post-exposure prophylaxis are reported in the United States (Brass, 1994; Fuenzalida, 1972). Post-exposure immunization is vital during the hours following contact with rabies, since it can prevent the disease that would otherwise cause the death of the patient (DeMaria, 2014; Mclean & WHO, 2011; Udow et al., 2013).

The estimated cost of this disease in Asia and Africa is $500 million dollars per year in direct costs, and more than $6 billion dollars in related costs, including loss of productivity, and the costs of vaccination and immunization (Knobel et al., 2005; Mclean & WHO, 2011). Currently, there is no effective treatment for rabies. For this reason, preventing the disease through vaccinations before and after exposure to the disease is a critical public health concern. In Ecuador, rabies vaccines continue to be produced using nerve tissue from suckling mice, principally for use in domestic animals. In other countries, these vaccines have been discontinued for human use because they are reactogenic and some are of low immunogenicity; instead of it, the use of the VERO cell rabies vaccine has been popularized.

In 1983 the first Meeting of the Directors of National Rabies Control Programs (in Spanish: Reunión de los Directores de los Programas Nacionales de Control de Rabia – REDIPRA) was held in Guayaquil, Ecuador, in response to recommendations by the 21^st^ Session of the Pan American Health Organization's (PAHO). As a result of the meeting, the strategies and the action plan for eliminating urban rabies from Latin America were approved, and since then the number of cases of urban rabies have declined by 95% (“Rabia,” 2014).

The aim of this study is to present the first synthesis of the epidemiology of rabies and its control efforts in Ecuador, in order to contribute to the public health management strategies in the country, given the lack of official national guidelines. We reviewed national and regional available data to describe the epidemiology of rabies in Ecuador, and present the current state of the rabies control efforts.

## 2. Methods

This observational study includes data from the Ecuadorian National Institute of Census and Statistics (INEC), mortality and morbidity data reported by the Ministry of Health and the National Institute for Social Security. The data of human rabies and dog rabies were analyzed using Pearson correlation in SPSS version 19.0 for Windows, and the maps were made using ArcMap version 10.1 for Windows.

Additionally, the study includes data collected from the production line of the different rabies vaccines of the National Institute of Public Health Research of Ecuador. Genetic diagnosis by RT-PCR and phylogenetic comparison of N genes from Challenge Virus Standard (CVS) vaccine strain used in Ecuador has been described previously (Nadin-Davis, Sheen, & Wandeler, 2009; Yockteng Melgar, 2014).

## 3. Results and Discussion

### 3.1 Human and Canine Rabies

In 1942 and 1943 the first documented outbreak of rabies was reported from the northern highland province of Carchi. The disease spread throughout the country over the next ten years, from Carchi to Imbabura provinces, followed by Cotopaxi, Manabí, Tungurahua (1945), Chimborazo and Bolivar (1947), Los Rios (1948), and Guayas provinces (1949). The southern provinces of the country, such as Loja, began reporting cases in 1954. There is no record of the first outbreak in the Amazon region (Ministerio de Salud Pública, 2014).

**Figure 1:**
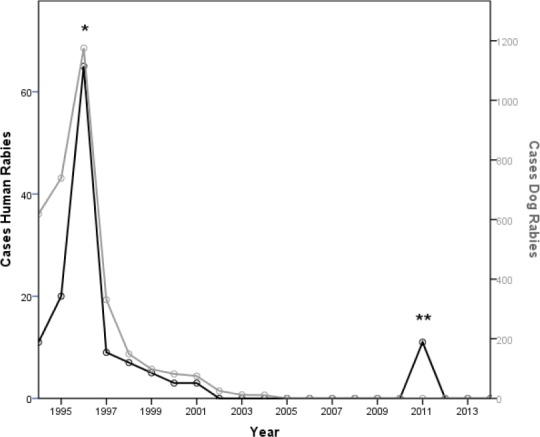
Annual reported cases of canine rabies (grey line) and human rabies cases (black line). *Start of national animal vaccination camping (1996). **Outbreak of sylvatic rabies due to transition by vampire bats (Desmodus rotundus) caused 11 human cases (2011). (Since 1994 to 2014, excluding 2011: Pearson correlation coefficient: r = 0.925, p<0.01)

**Figure 2:**
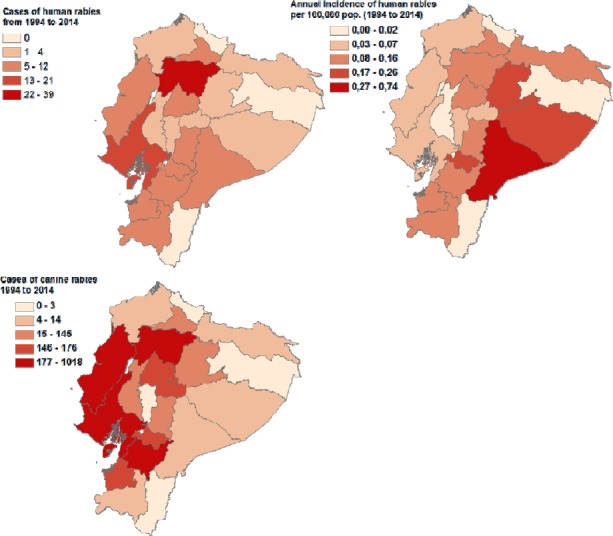
The spatial distribution of rabies by provinces in Ecuador. Top, clockwise: (1) total number of human rabies cases from 1994 to 2014, (2) the incidence of human rabies cases per 100 000 people per year from 1994 to 2014, and (3) the total number of canine rabies cases from 1994 to 2014

From 1994 to 2014, the overall change in human rabies cases can be attributable to dog rabies cases (Figure 1 and 2). This inference was supported by a Pearson correlation index of 0.925 (p<0.01). Data from 2011 was not included because that outbreak was attributed to vampire bats bites. In 1996 Ecuador suffered one of the worst rabies outbreaks in the last fifty years, due to the lack of effective rabies-control policies. Sixty-five human deaths caused by rabies were notified and 1175 cases of canine rabies reported in the same year. This was the highest rate of rabies per capita in the Americas in that year, resulting in 0.56 cases per 100 000 people per year (INEC, 2011).

Following the outbreak, the Ministry of Health intervened and effectively cut rabies transmission, leading to only 5 cases of human rabies and 98 cases of canine rabies by 1999. Interventions included mass vaccination campaigns of dogs, leading to over 80% of canine vaccine coverage, as well as the implementation of a public neutering program to control urban fauna. By 2001, rabies reports had reduced to three cases of human rabies and 75 cases of canine rabies (Figure 1). In 2004, the last cases of canine rabies were reported in the country and in 2009 the last cases of urban rabies in humans were reported in Esmeraldas province. Human rabies transmitted by dogs is close to being eradicated in Ecuador primarily due to dog vaccination campaigns, which provide an immune barrier to viral circulation.

In Latin America and the Caribbean, they have implemented similar plans that include: 80% coverage of vaccination; care 100% of people exposed; surveillance; and education about risk of rabies. This led to the entire region to a reduction of 25,000 cases in 1980 to fewer than 300 in 2010. Since 2004, sylvatic rabies has been the main concern in the region, since there are more cases of bat transmitted rabies than dog transmitted rabies. However, there are not effective methods to control rabies in this type of populations (Vigilato et al., 2013).

### 3.2 Sylvatic Rabies

There have been three important sylvatic rabies breakouts in the last 15 years. The first one occurred in 1997, when eight human deaths by rabies were confirmed in the Numbat-kaime and Kunkuki communities, located in Morona Santiago province. The second outbreak caused four deaths in 2005 in the Jatun Molino, Pastaza province. The last breakout of sylvatic rabies occurred in 2011 in Morona Santiago, within Wampuik, Tarimiat and Tsurik Nuevo communities resulting in 11 deaths, including 9 children under the age of 15. The transmission was alluded to vampire bats (*Desmodus rotundus*).

The Ministry of Health and the Ministry of Agriculture, Livestock and Fisheries joined efforts immediately to take control of the outbreak. The Ministry of Health provided 56 000 doses of prophylaxis rabies vaccines for people in the communities under risk. The Ministry of Agriculture, Livestock and Fisheries reduced vampire bat populations in rural areas through the use of diphenadione, an anticoagulant given to cattle, despite the ecological costs of killing bats (Ministerio de Salud Pública, 2014).

Apart from experiences during the recent outbreak, we have limited information on prevalence of vampire bat bites, the ecology and transmission dynamics of sylvatic rabies in Ecuador. A study of the community perception of bat bites in the Ecuadorian Amazon showed that over 20% of households heads reported being bitten within the last year, indicating a high risk of exposure (Romero-Sandoval, Escobar, Utzet, Feijoo-Cid, & Martin, 2014). Additional research is needed in this area.

### 3.3 Rabies Surveillance and Diagnostics

In Ecuador there is an epidemiologic surveillance system for rabies connected to the Regional Rabies Surveillance System in the Americas (SIRVERA), coordinated by PAHO. Local health units send a weekly report of positive cases of human and animal rabies cases. The cases must be confirm by a lab test. These reports are consolidated at national level and then sent to SIRVERA.

Rabies weekly reports include information on cases, treatment, laboratory diagnosis results, vaccination, and suspected animal cases. However, surveillance systems need to be strengthened to inform control interventions at the sub-country level, especially since most rabies cases are reported from rural areas, where underreporting is likely.

**Figure 3:**
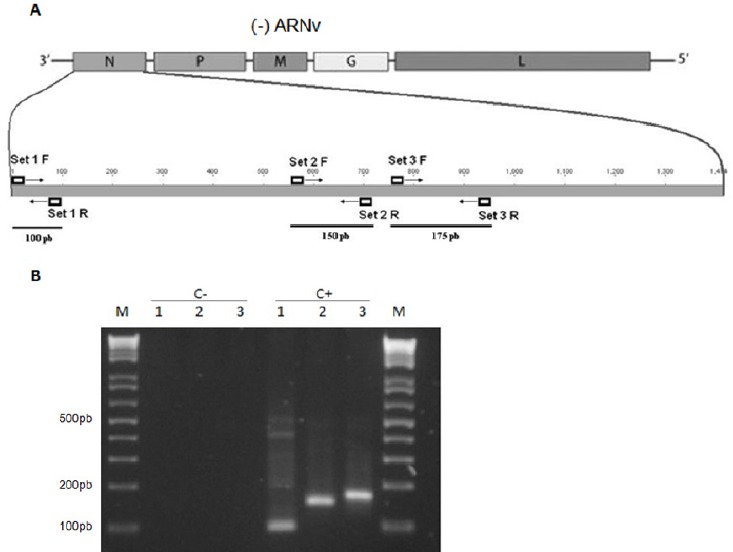
A) Schematic of the genetic organization of the rabies genome. The location of conserved regions for RT-PCR diagnosis is depicted. B) Agarose Gel resolving the RT-PCR diagnosis amplicons from three set of primers: 1, 2 and 3. Challenge Virus Standard (CVS) strain was use as the positive (C +) control and water as the negative (C-) control for RT-PCR. M is the DNA marker

The Ministry of Health has incorporated recently, with the help of Academia, a conventional RT-PCR method for rabies diagnostics, allowing for massive and independent processing of suspected samples (Yockteng Melgar, 2014). This was implemented by amplifying conserved regions of the nucleoprotein gene (Nadin-Davis et al., 2009) (Figure 3). Several reports have shown that genetic diagnosis is equal to or more sensitive and specific than the gold standard fluorescent antibody test (FAT) and mouse inoculation test (MIT) recommended by the WHO (Robardet, Picard-Meyer, Servat, & Cliquet, n.d.; Suin et al., 2014; Yang et al., 2012). Genetic methods can supplement diagnostic information generated by FAT and MIT tests to identify false negatives that result due to poor sample preservation, especially samples brought from remote communities (25). Several studies have shown that RT-PCR can detect genetic material even in decomposed or samples stored over long periods of time (Biswal, Ratho, & Mishra, 2007; Lopes, Venditti, & Queiroz, 2010).

**Figure 4:**
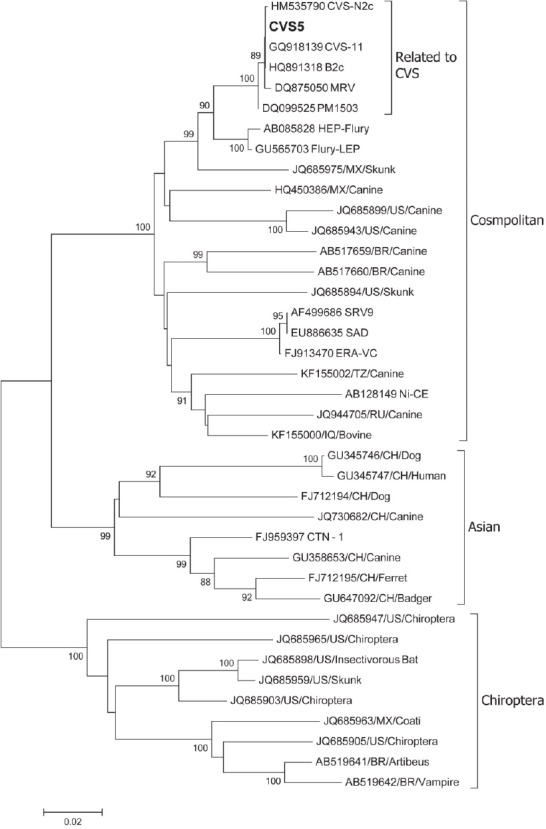
Phylogenetic comparison of N genes from Challenge Virus Standard (CVS) vaccine strain used in Ecuador (bold) with other rabies strains worldwide. The analysis was performed using Neighbour-Joining method based on the coding region of the N genes. Sequences were obtained from the GenBank. Bootstrap value >80% are shown next to the nodes

The implementation of the genetic diagnosis has led to a better characterization of rabies molecular epidemiology in Ecuador. The phylogenetic relationship of the N gene from the Challenge Virus Standard (CVS) rabies virus strain, used in Ecuador for vaccine production, aligned with the Cosmopolitan lineage and Asian strains, the latter being the source of most urban (canine) rabies (Figure 4). However, all reported recent rabies outbreak have been associated with Chiroptera (bats) which segregated to a different clade, indicating genetic drift from the vaccine and a probable impairment in immunologic protection.

### 3.4 Rabies Vaccine Manufactured in Ecuador

In Ecuador, the current vaccine manufacturing process is based on the Fuenzalida-Palacios technique, which uses baby mouse brain infected with the rabies virus. Vaccines produced by this process are characterized by a high antigenic potency and an induced quick-lasting serologic response (Rosario Z. de Dávila et al., 1988). Human-use rabies vaccines are formulated using a UV-treated 2% baby mouse brain suspension from 24-hour-old mice that are previously inoculated with CVS 51 and 91 rabies virus strains. This vaccine has a 24-month shelf life when maintained at 2 to 8 ºC without light exposure. All manufacturing process, procedures and techniques are in accordance with standards set by WHO/PAHO (Horowitz et al., 2003; “WHO | Rabies,” n.d.).

Vaccine quality is ascertained through official release of each production batch, after all quality controls have been passed, with normalized procedures described in the Manual: Rabies-Lab techniques of the WHO (Meslin, Kaplan, Koprowski, Organization, & others, 1996). The vaccine potency test of the National Institute of Health (NIH test) is also performed, as described in the US Code of Federal Regulations (9CFR 113.209) (U.S. Government, 2014).

Over the last 20 years in Ecuador, 2 214 800 doses of nationally produced rabies vaccine were administered to people. In 2012, Ecuador discontinued domestic production of human rabies vaccines due to the implementation of a new administrative structure at the Ministry of Health, and began importing inactivated rabies vaccine grown in Vero cells. Canine vaccine is the only animal rabies vaccine still manufactured in Ecuador.

The effectiveness of the human and canine rabies vaccine made in Ecuador has never been assessed; however, the decline in the incidence of rabies in the country following mass campaigns is indicative of its effectiveness (Figure 1). Whilst in Ecuador there have not been reported severe adverse events, it is necessary to implement an adequate post-commercial control system. However, as an indicator of vaccine safety, a study of Ecuadorian manufactured vaccine was performed by the Ecuadorian NIH and found that adverse events in people exposed to the Ecuadorian rabies vaccine did not exceed international standards established for this product (Rosario Z. de Dávila et al., 1988). Consistency of manufacturing quality has been judged by the fact that almost all batches (99%) of vaccine that were produced were released to the public for human use.

In 2009, by presidential decree, the pharmaceutical state company (ENFARMA) was created, and it assumed the vaccine production competencies that were performed previously by the Ecuadorian NIH. This public company has the task of providing medicine to the National Health system. ENFARMA established a plan to implement a relative new technology (VERO cell cultures) for rabies vaccine production in new manufacturing facilities in the country. The company anticipates an annual production of 100 000 of human-use and 30 000 of veterinary-use doses. The re-establishment of local production will be important to control rabies outbreaks; however, vaccine production has been delayed until the construction of new manufacturing plants is completed in 2016.

## 4. Conclusion

This study provides the first compilation of the epidemiological data on rabies in Ecuador and a review of control efforts to date. Human and canine rabies, also known as urban rabies, have clearly decreased and are near eradication as the result of massive canine vaccination campaigns. High rates of canine vaccine coverage; as well as the implementation of sanitary measures for controlling urban fauna, have achieved the reduction of transmission from urban fauna to humans in Ecuador. These measures were implemented mainly after the epidemic in 1996. These strategies have decreased human rabies in many countries in South America (INEC, 2011; Paredes Cecilia, 2010), resulting in complete eradication in other countries (Smith, Orciari, Yager, Seidel, & Warner, 1992).

Sylvatic rabies remains a prevalent public health threat in Ecuador and other countries in the Amazon region. Our phylogenetic study (13) revealed that the local vaccine strain differs from the clade of sylvatic rabies, implying a different amino acid sequence that might affect the effectiveness of the vaccine. However, further antigenicity and immunogenicity studies must be done to assess the effectiveness of the local vaccine against sylvatic rabies strains. Additionally, we suggest the implementation of molecular surveillance of sylvatic rabies to better understand the virus evolution in Ecuador.

The public health sector should focus its efforts to educate people such as rural health workers and farmers, who may be in risk of rabies exposure. Multi-country collaborations for sylvatic rabies control are especially important in the Amazon region, where there is a high risk of cross-border disease propagation (Blanton et al., 2011; Takayama, 2008).
